# Trade-off between constitutive and inducible resistance against herbivores is only partially explained by gene expression and glucosinolate production

**DOI:** 10.1093/jxb/erv033

**Published:** 2015-02-25

**Authors:** Sergio Rasmann, Estelle Chassin, Julia Bilat, Gaétan Glauser, Philippe Reymond

**Affiliations:** ^1^Institute of Biology, University of Neuchâtel, 2000 Neuchatel, Switzerland; ^2^Department of Ecology and Evolution, University of Lausanne, Biophore building, 1015 Lausanne, Switzerland; ^3^Neuchâtel Platform of Analytical Chemistry, University of Neuchatel, 2000 Neuchatel, Switzerland; ^4^Department of Plant Molecular Biology, University of Lausanne, Biophore building, 1015 Lausanne, Switzerland

**Keywords:** Glucosinolates, jasmonic acid, plant defences, plant–herbivore interaction, specificity of resistance, VSP2

## Abstract

The hypothesis that constitutive and inducible plant resistance against herbivores should trade-off because they use the same resources and impose costs to plant fitness has been postulated for a long time. Negative correlations between modes of deployment of resistance and defences have been observed across and within species in common garden experiments. It was therefore tested whether that pattern of resistance across genotypes follows a similar variation in patterns of gene expression and chemical defence production. Using the genetically tractable model *Arabidopsis thaliana* and different modes of induction, including the generalist herbivore *Spodoptera littoralis,* the specialist herbivore *Pieris brassicae*, and jasmonate application, constitutive and inducibility of resistance was measured across seven *A. thaliana* accessions that were previously selected based on constitutive levels of defence gene expression. According to theory, it was found that modes of resistance traded-off among accessions, particularly against *S. littoralis,* in which accessions investing in high constitutive resistance did not increase it substantially after attack and vice-versa. Accordingly, the average expression of eight genes involved in glucosinolate production negatively predicted larval growth across the seven accessions. Glucosinolate production and genes related to defence induction on healthy and herbivore-damaged plants were measured next. Surprisingly, only a partial correlation between glucosinolate production, gene expression, and the herbivore resistance results was found. These results suggest that the defence outcome of plants against herbivores goes beyond individual molecules or genes but stands on a complex network of interactions.

## Highlight

The observed partial correlation between herbivore resistance, defensive metabolites accumulation, and gene expression suggests a complex network of gene interactions governing the postulated trade-off between constitutive defences and their inducibility.

## Introduction

Plants, to ward off herbivore attack, have evolved a whole array of defence traits (Schoonhoven *et al.*, 2005), which can always be present or only induced after herbivore feeding (Karban and Baldwin, 1997). The general consensus argues that inducible defences have evolved as a cost-saving strategy (Karban *et al.*, 1997), in which undamaged plants can divert resources from defence to growth and reproduction. Zangerl and Rutledge (1996) postulated that the pattern of constitutive and inducible defences, at the plant or at the organ level, depends on the probability of the attack and the value of the organ. In other words, plants or organs that are regularly attacked by herbivores, should have high levels of constitutive defences and low levels of induced defences. By extrapolations, in populations where herbivory is low, plants should invest little in constitutive defences and more in inducibility of defence, in which inducibility is the difference between the induced state minus the constitutive state of defence in an organ of the plant. Recent examples have shown that inducibility is dependent on the spatial variation of the plant populations and herbivore pressure (Moreira *et al.*, 2014; Rasmann *et al.*, 2014), suggesting that, at the landscape level, there are constraints on simultaneously producing both types of defence investment within one species.

Indeed, because it is known that the expression of redundant traits is costly for the plant (Koricheva *et al.*, 2004), and because it is assumed that constitutive and induced defences are two traits in competition for the same resources in the plant, a trade-off (or negative correlation) should be expected between them (Agrawal *et al.*, 2010). In other words, if both constitutive and inducible resistance traits are adaptive, a negative correlation should be observed between constitutive and induced resistance across populations or species of plants (Agrawal *et al.*, 2010). Several examples have shown trade-offs between constitutive and inducible resistance, both within (Gianoli, 2002; Rasmann *et al.*, 2011, 2014) and across species (Zhang *et al.*, 2008; Kempel *et al.*, 2011; Moreira *et al.*, 2014; Rasmann and Agrawal, 2011). In addition, Thaler and Karban (1997) mapped constitutive and inducible defences along the phylogeny of *Gossypium* spp., and showed independent and repeated origins and losses of both defence traits, indicating evolutionary lability and independence in the mode of defence investment. In *Acacia*, it was shown that constitutive extrafloral nectar production originated from inducible production in closely related species (Heil *et al.*, 2004). To summarize, past research indicates that constitutive and inducibility of resistance evolve depending on the herbivore pressure and the probability of attack at a particular site. Nevertheless, constraints imposed by resource acquisition force the two modes of defence investment to correlate with each other negatively.

With this study, the aim was to take a step further in the study of the interactions, and putative trade-offs, between inducible and constitutive resistance and to investigate the genetic bases explaining the pattern. The question specifically asked was whether patterns of trade-off between constitutive and inducible resistance [i.e. the effect of the plant’s defensive arsenal on the performance of the herbivores, according to Karban and Baldwin (1997)] is correlated to similar patterns of defensive secondary metabolites and gene induction. To address these questions, a highly genetically-tractable plant was used, the thale cress *Arabidopsis thaliana* (Brassicaceae) which is a small annual plant from Eurasia but naturalized across all continents except Antarctica. Basal genome-wide expression levels have been characterized for many *Arabidopsis* accessions. In addition, major biosynthetic pathways involved in insect resistance, including the jasmonate pathway (Howe and Jander, 2008), are well characterized (Bodenhausen and Reymond, 2007). Furthermore, *Arabidopsis*, like most species in the Brassicales, contains glucosinolates. When insect herbivores feed on the plant, they damage tissues and bring glucosinolates in contact with an activated enzyme, the myrosinase, which results in the production of highly toxic hydrolysis breakdown products such as nitriles, isothiocyanates or thiocyanates (Halkier and Gershenzon, 2006). Moreover, several studies have already shown specificity in inducible resistance against specialists versus generalist herbivores in *Arabidopsis* (De Vos *et al.*, 2005; Rasmann *et al.*, 2012). Generally, it was shown that the glucosinolates have a negative impact on generalist herbivore fitness, but it has little, none or a positive effect on specialist herbivores (Mueller *et al.*, 2010; Schweizer *et al.*, 2013).

It was hypothesized here that (i) according to classic theory, previously induced plants are more defended against subsequent herbivore attack than undamaged plants; (ii) generalist herbivores are more susceptible than specialist herbivores, (iii) there is a negative genetic correlation between constitutive and inducibility of resistance, and (iv) both glucosinolate production, and gene expression related to defence induction, correlate with patterns of induced resistance.

## Materials and methods

### Plant material

Seeds of all accessions were obtained from The Nottingham Arabidopsis Stock Centre (NASC). For all the experiments (see below), all plants were grown in a growth chamber (short days, 20 °C, 55% RH) with a 3:1 v/v mix of commercial potting soil (Orbo-2, Schweizer AG, Lausanne; Switzerland) and perlite. All plants were 6-weeks-old at the time of the experiments.

### Microarray data

Constitutive expression data for the *Arabidopsis* accessions were downloaded from the ArrayExpress repository database (http://www.ebi.ac.uk/arrayexpress; experiment E-TABM-18). Data are part of the At GenExpress project (http://arabidopsis.org/portals/expression/microarray/ATGenExpress.jsp) and consist of expression values from 4-d-old seedlings from 34 accessions grown in soil in the same conditions and at the same time (Lempe *et al*, 2005).

### Inducible resistance experiment

To measure the specificity of trade-offs between inducible and constitutive resistance in *Arabidopsis*, an experiment with three different induction treatments was performed: a control treatment (no induction), a jasmonic acid (JA) application, and a herbivore induction. Jasmonic acid has been shown to be the master regulator of plant inducible resistance against chewing herbivores in many plants, including *Arabidopsis* (Howe, 2004; Howe and Jander, 2008). For the herbivore treatment, the highly generalist herbivore *Spodoptera littoralis* (Lepidoptera, Noctuidae) and the cabbage family specialist herbivore *Pieris brassicae* (Lepidoptera, Pieridae) were chosen. Eggs of *S. littoralis* were provided by Syngenta (Stein Switzerland) and first-instar larvae were obtained by placing eggs at 30 °C for 3 d. First-instar larvae of *P. brassicae* were obtained from rearing insects on cabbage (*Brassica oleracea*) in controlled greenhouse conditions at the University of Lausanne.

For all treatments, plants were enclosed in hermetic Plexiglas boxes (*n*=7 genotypes×3 treatments×2 herbivores×3 plants=63 plants). Treatments were performed as follows: (i) the control plants were left without further treatment for 3 d; (ii) the JA treatment included plants that were induced by putting three cotton buds in the box, each one spiked with 5 µl of methyl jasmonate (MeJA) (Sigma-Aldrich CAS Nb 39924-52-2). JA treatment lasted 24h after which lids were opened to allow the evaporation of the JA left in the box. Finally, (iii) plants were induced by placing 8–10 first-instar *S. littoralis* larvae per pot. Larvae were allowed to feed for 3 d prior to removal. *S. littoralis* was used for the induction treatment as this herbivore was used to measure the induction of defence genes in selected accessions (see below).

After the induction, plants were individually surrounded with 330ml volume deli plastic cups with the bottom cut off, and 10 *S. littoralis* or 10 *P. brassicae* larvae were added to each plant (*n*=30 larvae per herbivore, per genotype, and per treatment). Cups were covered with fine-meshed nylon nets to prevent larvae from escaping, and larvae were allowed to feed for 7 d, after which, all surviving larvae were flash-frozen in liquid nitrogen, oven-dried for 4 d at 50 °C, and weighed.

### Glucosinolate and gene expression analyses

For glucosinolate and gene analyses, 12 plants per genotype were planted and, after 6 weeks, half of the plants were induced with 10 *S. littoralis* caterpillars for 3 d as described above. At the end the induction treatment, 200mg of fresh tissue per plant was ground with a homogenizer in 2ml ice-cold MeoH:water (70:30, v/v) with 25 μl of sinalbin 1.56 mmol as the internal standard. Samples were then incubated for 15min at 80 °C in a block heater (Techne dri-block, Staffordshire, UK), centrifuged at 3500×*g* for 10min, and the supernatant was transferred to an appropriate vial for analysis. Glucosinolate identification and quantification was performed using an Acquity UPLC from Waters (Milford, MA, USA) interfaced to a Synapt G2 QTOF from Waters with electrospray ionization, using the separation and identification method as described in Glauser *et al.* (2012).

For gene expression analyses, two leaves were sampled from half of the control and treated plants (*n*=3), added together in one Eppendorf tube and flash-frozen in liquid nitrogen. Three genes known to be induced after caterpillar attack in Col-0 were selected (Reymond *et al.*, 2000), including: (i) *ALLENE OXIDE CYCLYSE2* (*AOC2*), a gene that catalyses an essential step in jasmonic acid biosynthesis; (ii) *VEGETATIVE STORAGE PROTEIN2* (*VSP2*), a highly inducible gene after herbivory or JA treatment; and (iii) *CYTOCHROME P450 79B3* (*CYP79B3*), a gene involved in indole-glucosinolate biosynthesis. RNA extraction and qPCR analyses were done following standard protocols using the reference gene *At2g28390* (*Arabidopsis* SAND family protein) as described in Hilfiker *et al.* (2014). Primer efficiencies (*E*) were assessed by a five-step dilution regression (see list of primers in Supplementary Table S5 at *JXB* online). The expression level of a target gene (TG) was normalized to the reference gene (RG) and calculated as Normalized Relative Quantity (*NRQ*) as follows: *NRQ*=*E* ^CtRG^/*E* ^CtTG^.

### Statistical analyses

The effect of the genotypes, the induction treatment, and the two herbivore species was analysed using a full-factorial three-way ANOVA. Secondly, to test for trade-offs between constitutive and inducibility of resistance, the inducibility (i.e. the difference in mean larval mass values for each genotype between control and induced plants) was regressed against the genotype mean of that trait in the control treatment (i.e. the constitutive level). As a variable was regressed against a difference that includes the same variable (i.e. inducibility of resistance=induced plants–control plants), the errors in the two axes are not independent, and so there is the possibility of obtaining spurious correlations from these analyses (Morris *et al.*, 2006). Therefore, in order to evaluate the significance of these correlations, the Monte Carlo simulation procedure proposed by Morris *et al.* (2006) was employed using MATLAB (Version 7.5.0.342 – R2007b, MathWorks Inc., USA).

Glucosinolate data were analysed with a three-way permutation ANOVA using the package LmPerm in R (Wheeler, 2010) because it was not possible to reach normality of the errors, and included genotype, herbivore treatment, and compound identity as the main effects.

## Results

### Selection of *Arabidopsis* accessions with contrasting constitutive defences

To investigate the genotypic variation in constitutive versus inducible resistance, seven accessions of *Arabidopsis* were selected based on the expression of 16 genes known to be related to defence against chewing herbivores (Reymond *et al.*, 2004; see Supplementary Table S1 at *JXB* online). For each individual gene, 34 accessions for which whole-genome expression data were available (see the Materials and methods) were ranked based on the constitutive expression of defence genes. The computation of the average constitutive expression across all genes provided a list of seven accessions (see Supplementary Table S2 at *JXB* online), including HR-5, Kindalville-0 (Kin-0), Niederzenz-1 (Nd-1), Columbia-0 (Col-0), Moscow-0 (Ms-0), C-24, and Shahdara (Sha).

### Induction experiment

**Fig. 1.**
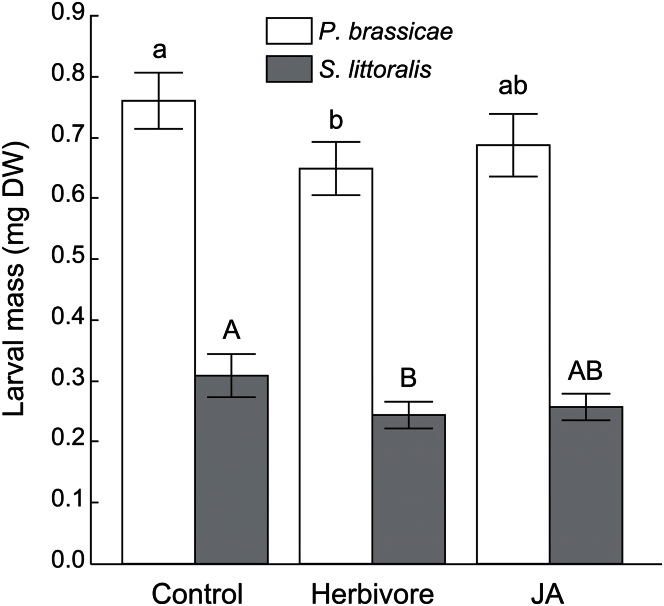
Induced resistance against chewing herbivores. Shown are means (±SE) of *P. brassicae* (open bars) and *S. littoralis* (shaded bars) larval mass on *Arabidopsis* plants that were either left untouched (control), previously induced with *S. littoralis* caterpillars or previously induced with methyl jasmonate (JA). The average of resistance across seven *Arabidopsis* accessions is shown. Different letters above the bars means a difference after the post-hoc Tukey test, *P* <0.05.

In accordance with classic predictions, an overall effect of previous induction on resistance was found (Fig. 1; Table 1). In particular, larvae of both species grew 22% and 14% less (for *S. littoralis* and *P. brassicae*, respectively) on plants that had previously been induced by *S. littoralis* (Fig. 1; Table 1), and to a lesser extent on plants that were induced with JA (17% and 10%, respectively, see no effect of treatment×species interaction in Table 1). Overall, strong variation in resistance was found across accessions (Table 1) and strong specificity in resistance across accessions (see significant genotype×species interaction in Table 1).

**Table 1.** Three-way ANOVA for assessing the effect of the seven *Arabidopsis* accessions, the induction treatment (with *S. littoralis* or with methyl jasmonate), on the growth the two herbivore species (*S. littoralis* and *P. brassicae*)

Factor	df	*F* ratio	*P* value
Genotypes (G)	6	5.646	<0.0001
Treatments (T)	2	3.999	0.022
G×T	12	1.400	0.183
Species (S)	1	261.774	<0.0001
G×S	6	3.354	0.005
T×S	2	0.214	0.807
G×T×S	12	1.327	0.220
Residuals	82		

**Fig. 2.**
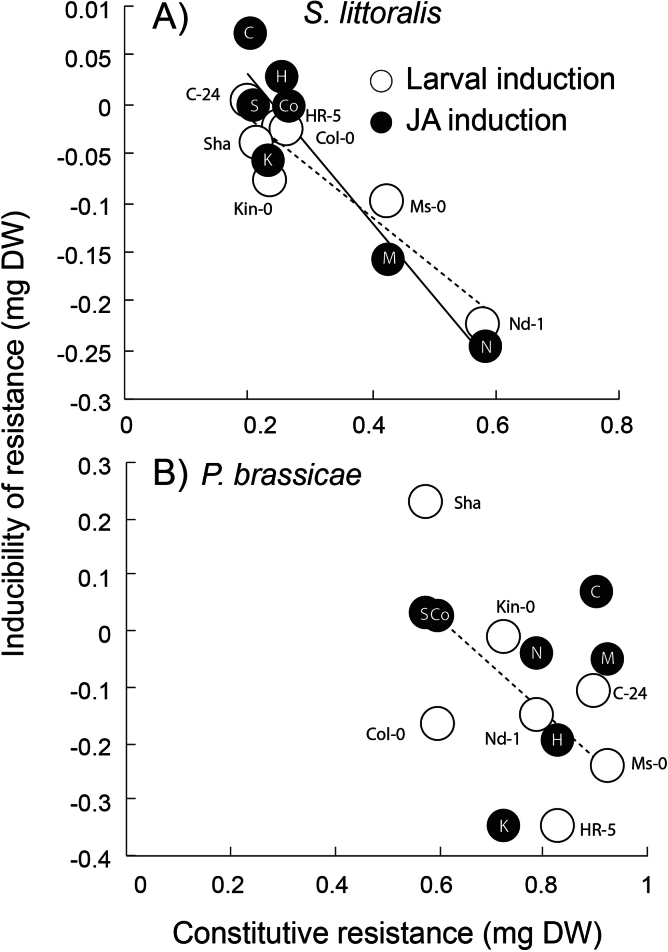
Trade-off between constitutive and inducibility of resistance. The means of (A) *S. littoralis* and (B) *P. brassicae* larval mass when feeding on seven *Arabidopsis* accessions is shown. Plants were either left undamaged (constitutive) or previously induced by herbivores (open circles, dotted lines), or induced with methyl jasmonate (black dots, solid lines). Inducibility is the average difference of larval weight between the induced and constitutive conditions and, therefore, a negative value means induced resistance and the lowest values indicate the highest induction of resistance. Lines indicate a significant correlation, *P* <0.05. Letters next to the open circles or inside the black circles indicate the accessions’ names: N=Nd-1, M=Ms-0, K=Kin-0, S=Sha, Co=Col-0, H=HR-5, and C=C-24.

Across seven accessions of *Arabidopsis*, a negative genetic correlation was found between the constitutive resistance and the inducibility of resistance, particularly for the generalist herbivore *S. littoralis* (Fig. 2; for *S. littoralis*, larval induction, *r*= –0.94, *P*=0.02; and JA induction, *r*= –0.94, *P*=0.01; and for *P. brassicae*, larval induction, *r*= –0.82, *P*=0.09; and JA induction, *r*= –0.08, *P*=0.74). For *S. littoralis*, the ranking of inducibility from highly induced susceptibility to highly induced resistance for both the larval and the jasmonate induction was: C-24, HR-5, Sha, Col-0, Kin-0, Ms-0, and Nd-1. In other words, Nd-1 showed the largest inducibility of resistance, whereas C-24 had the smallest. Interestingly, in some instances, it was observed that larvae were larger on induced plants than on uninduced ones (see Supplementary Table S3 at *JXB* online). This was the case for *S. littoralis* feeding on HR-5 and C-24 after treatment with JA, and for *P. brassicae* feeding on Sha and C-24, after herbivory.

**Fig. 3.**
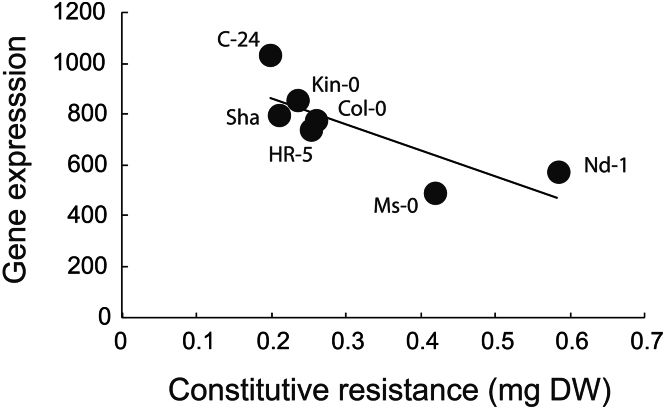
The relationship between constitutive gene expression and resistance against chewing herbivores. The genotypic relationship across seven *Arabidopsis* accessions of resistance against *S. littoralis* larvae and the average gene expression of eight genes related to glucosinolate production is shown (*P* <0.05).

It was then assessed whether natural variation in gene expression could directly influence resistance. Therefore, the average expression values of eight genes related to glucosinolate production and eight genes including JA marker genes and JA biosynthesis in *Arabidopsis* (see Supplementary Table S2 at *JXB* online) were regressed against the larval weight of the generalist *S. littoralis* on each genotype (see Supplementary Table S3 at *JXB* online). Only *S. littoralis* data were used for this analysis since only generalist herbivores should be affected by glucosinolates in plants. In addition, only the control treatment was used as gene expression was measured on undamaged plants. It was found that the constitutive expression of glucosinolate biosynthesis-related genes negatively predicted larval weight gain (Fig. 3; *n*=7, *r*=0.80, *P*=0.03). This was not true when regressing the average expression of genes related to JA signalling and production (*n*=7, *r*=0.07, *P*=0.87). To test whether or not results for the glucosinolate genes were spurious due to random gene sampling, a permutation analysis was performed using the 10 000 averages of 10 randomly selected genes from the whole pool of 22 759 genes present in *Arabidopsis*. As shown in Supplementary Fig. S1 at *JXB* online, our data indicate that the glucosinolate result is well below the 0.1 and the 0.05 probabilities when compared with correlations with random genes, indicating that the *S. littoralis* result cannot be obtained from random gene sampling of defence genes.

### Glucosinolate and gene expression analyses

**Fig. 4.**
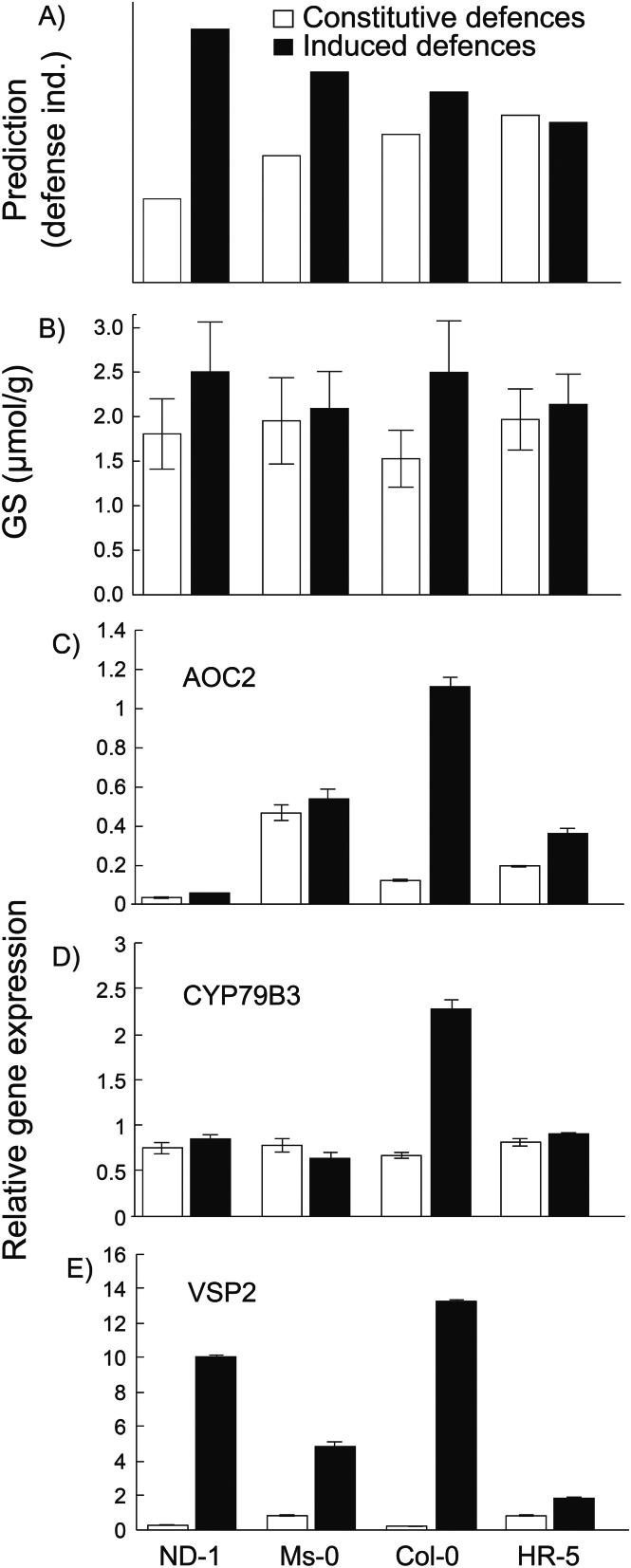
Defence induction across accessions. (A) The predicted defence induction of four *Arabidopsis* accessions based on the resistance bioassay in Fig. 2A, in which Nd-1 should have the highest inducibility, HR-5 and Col-0 should have the lowest inducibility and Ms-0 should have an intermediate level of inducibility.(B) The mean (±SE) levels of constitutive (open bars) and induced (black bars) production of glucosinolates is shown and (C–E) show the relative expression of *AOC2*, *CYP79B3*, and *VSP2*, respectively. Induction was performed with *S. littoralis* caterpillars. Values (±SE) are the average of three technical replicates.

Because a negative relationship was observed between constitutive and inducible resistance (particularly against *S. littoralis*), an attempt was made to find the defence mechanisms behind the observed trade-off and so glucosinolates and gene expression of Col-0, HR-0, Ms-0, and Nd-1 were measured. The initial results from the resistance experiment indicated that Col-0 and HR-5 showed little or no induced resistance, Ms-0 showed intermediate levels of induced resistance, and Nd-1 showed the highest levels of induced resistance (Fig. 2A). It was therefore predicted that glucosinolate and gene expression profiles would mimic the larval resistance results, and Nd-1 would show the highest induction of defensive metabolites and genes related to defence induction, and Col-0 and HR-5 the lowest (Fig. 4A).

Glucosinolate analyses yielded 14 individual glucosinolate compounds, all showing different overall levels (see Supplementary Table S4 at *JXB* online; see the compound effect in Table 2) and different inducibilities after herbivore attack (see the treatment×compound effect in Table 2), overall, with herbivore treatment increasing average glucosinolate levels by 27% compared with control plants (see the treatment effect in Table 2). Accessions showed little variation in total amount of glucosinolates, and only Nd-1 and Col-0 showed variation in glucosinolate induction after herbivore attack (Fig. 4B; see Supplementary Table S4 at *JXB* online; see the treatment×genotype interaction in Table 2). Strikingly, some glucosinolates were almost exclusively found in a single accession (see Supplementary Table S4 at *JXB* online).

**Table 2.** *Three-way permutation ANOVA table for individual glucosinolate levels across four* Arabidopsis *accessions* Plants were either left undamaged or induced with *S. littoralis* caterpillars for 3 d (i.e. treatment effect).

Factor	Df	Iter	*P* value
Genotype (G)	3	51	1
Treatment (T)	1	3985	0.024
G×T	3	3026	0.032
Compound (C)	13	5000	<0.0001
G×C	39	5000	<0.0001
T×C	13	5000	0.025
G×T×C	39	5000	0.004
Residuals	560		

Expression analyses of selected insect-inducible genes showed strong induction after *S. littoralis* treatment (Fig. 4C–E; Table 3). *VSP2* had the highest inducibility, with 14-fold induction overall (Fig. 4E), compared with 2.6-fold and 1.55-fold for *AOC2* and *CYP79B3* (Fig. 4C, D, respectively). A strong genotype effect and a genotype×treatment effect was also found for the inducibility of genes (Table 3). For *VSP2*, Col-0 and Nd-1 showed the strongest induction, MS-0 showed average induction, and HR-5 the lowest induction after herbivore attack. However, *AOC2* was strongly induced in Col-0, moderately in both HR-5 and Nd-1, but not in Ms-0. Finally, *CYP79B3* was only induced in Col-0 (Fig. 4D). Since this enzyme is involved in the synthesis of indole-glucosinolates (Halkier and Gershenzon, 2006), and its expression correlates with accumulation of glucosinolates in Col-0 (Schweizer *et al.*, 2013), it was interesting to see that levels of the main indole-glucosinolates I3M, and to a lesser extent 1MOI3M, increased in Col-0 after herbivory (see Supplementary Table S4 at *JXB* online). In addition, both compounds were also induced in Nd-1 and I3M was higher in Ms-0 without the respective changes in *CYP79B3* expression. Thus, our data show that there is not a consistent correlation between inducibility of resistance, accumulation of glucosinolates, and defence gene induction between accessions as predicted by the model in Fig. 4A.

**Table 3.** Three-way permutation ANOVA table for individual gene expression levels across four *Arabidopsis* accessions Plants were either left undamaged or induced with *S. littoralis* caterpillars for 3 d (i.e. treatment effect).

Factor	Df	Iter	*P* value
Genotype (G)	3	5000	<0.0001
Treatment (T)	1	5000	<0.0001
G×T	3	5000	<0.0001
Genes (Gn)	2	5000	<0.0001
G×Gn	6	5000	<0.0001
T×Gn	2	5000	<0.0001
G×T×Gn	6	5000	<0.0001
Residuals	48		

## Discussion

It was found that overall inducible resistance against herbivores in *Arabidopsis* is underlined by strong genotypic variation, in which accessions that have high constitutive resistance are weak inducers, whereas accessions that have low constitutive resistance are strong inducers. This pattern generates the predicted trade-off between constitutive and inducible resistance in plants. Interestingly, despite the fact that the basal expression of genes related to glucosinolate biosynthesis also predicts the observed resistance to herbivory, it was found that constitutive and induced glucosinolate levels and defence gene induction only partially relate to the observed resistance. This suggests that plant defence allocation strategies goes beyond the individual molecules or genes but stands on a complex network of interactions. The possible causes and consequences of the observed results are discussed below.

### Specificity of induction of defences and herbivore responses

The seminal book on plant defence induction by Karban and Baldwin (1997) has paved the way to the general wisdom that plants, under herbivore attack, are able to increase their basal levels of defences to a higher level. Whereas the ability to increase resistance only after attack has undoubtedly clear benefits in term of costs (Karban *et al.*, 1997), several drawbacks still impair a full grasp of the phenomenon, including high specificity on the induction/response, and strong genotypic variation in induction.

First, as shown here, there is high level of specificity on both sides, in which either the induction agent (an insect or a phytohormone in our case) can result in different inducibilities and the response of the herbivore is species specific. Indeed, plant induction of defences is driven by the complex chemistry of plant–herbivore interaction (Walling, 2000; Halitschke *et al.*, 2003), which takes into account the counter-response of the herbivore (Felton and Eichenseer, 2000; Karban and Agrawal, 2002), and surely goes beyond simple application of jasmonic acid to the plant (but see Rasmann *et al.*, 2012). Therefore, only by studying the effect of several inducing agents can we generalize on the existing patterns. Next, it is shown that specialist herbivores such as *P. brassicae* are less affected by previous plant induction than the generalist herbivore *S. littoralis* and this seems to be a general rule in plant–insect interaction studies (Ali and Agrawal, 2012). Whether variation in induced resistance and the subsequent formation of trade-offs is mainly generated by generalist herbivores is an enticing question, and to our view merits further studies.

Second, this is not the first example of genotypes becoming more susceptible to herbivores after induction. Indeed, induced susceptibility is more common than we might expect (Karban and Baldwin, 1997), and it has been suggested that defence suppression could even benefit the plant rather than the herbivore (Kahl *et al.*, 2000). Although there is generally still little evidence for it, other studies show that plants decrease their defences (Kahl *et al.*, 2000; Bede *et al.*, 2006; Lawrence *et al.*, 2008), and become more susceptible to attacks by herbivores after previous attacks by other species of herbivores (Sauge *et al.*, 2006; Poelman *et al.*, 2008; Sarmento *et al.*, 2011). Mechanisms behind induced susceptibility might include trade-offs between defence types against different herbivore species, via so-called antagonistic cross-talk between signalling pathways involved in plant defence (Thaler, 1999), even within the same species (Bruessow *et al.*, 2010). It is, therefore, possible that the physiological (and evolutionary) constraints generating the trade-offs between constitutive and inducibility of resistance might also be behind patterns of induced susceptibility, and future work with *Arabidopsis* in this regard might answer this question.

### Genetic correlations among resistance strategies

By measuring caterpillar growth on undamaged and previously damaged plants, a negative genetic correlation was found between constitutive resistance and inducibility of resistance. Thus, *Arabidopsis* accessions appear to have a maximal potential for resistance, and this is either allocated constitutively (i.e. always present) following herbivore attack or in equal balance between the two. Such trade-offs between constitutive and induced responses suggests that the expression of resistance traits in plants is costly or otherwise constrained, or that there is simply no benefit in additional resistance beyond a particular threshold level (Agrawal *et al.*, 2010). Similar patterns in deployment strategies of defence were previously observed within genotypes (Rasmann *et al.*, 2011), or across species of plants (Kempel *et al.*, 2011; Moreira *et al.*, 2014). Nevertheless, others have failed to observe trade-offs between constitutive defences and inducibility, at least across species (Rasmann and Agrawal, 2011). Such discrepancies in the experimental observations are difficult to explain as long as a mechanistic understanding of how trade-offs arise, particularly at the gene level, is lacking (Agrawal *et al.*, 2010). As mentioned above, variable production of defences can be triggered by insect-derived elicitors (Halitschke *et al.*, 2003), plant hormones (Harfouche *et al.*, 2006), herbivore-induced volatile organic compounds (Ton *et al.*, 2007) or, indeed, differential constitutive levels of gene expression (Ahmad *et al.*, 2011).

In addition, differential investment in plant defence deployment could arise from different herbivore pressures across the effective niche distribution of the species. For instance, it has recently been shown that *Vicia sepium* plants at high elevation have lower basal levels of volatile organic compounds production but are more inducible than their conspecifics at lower elevations. This pattern of defence deployment goes hand-in-hand with lower herbivore pressure and a lower abundance of predatory ants at high elevation (Rasmann *et al.*, 2014). It is therefore suggested that the observed pattern in *Arabidopsis* accessions is generated both by the physiological constrains of the plant (i.e. some genotypes are simply at the maximum level of resistance and thus could not be induced even more as was shown in Córdova-Campos *et al.*, 2012), and the different selection pressures at different locations where the accessions originated.

### Genotype–phenotype correlations

Contrary to our expectations, a consistent correlation was not observed between the phenotypic response (i.e. herbivore growth), glucosinolate production, and defence gene induction. For instance, although the increasing induction of VSP2 between HR-5, Ms-0, and Nd-1 was correlated with the inducibility of resistance results (as predicted in Fig. 4A), Col-0 displayed the strongest induction of defence genes and it displayed a high constitutive defence. Similarly, accumulation of glucosinolates after *S. littoralis* feeding was not higher in Nd-1 than Col-0, despite their different inducibility of resistance. In addition, the constitutive expression level of glucosinolate biosynthesis genes was negatively correlated with larval weight, although this was not true for glucosinolate levels, implying another level of complexity. In a related study with *Arabidopsis*, Ahmad *et al.* (2011) showed that a high induction of the defence gene *PR1* was correlated with a reduced bacterial infection in different accessions.

Clearly, more work is needed to understand these discrepancies better. For example, the apparent absence of correlation between total glucosinolates levels and the inducibility of resistance might be explained by the fact that different accessions contain specific glucosinolates. These molecules may have different deterrent properties and a careful examination of the contribution of each glucosinolate compound to defence will be needed. Furthermore, our investigation was restricted to genes of the jasmonate pathway and to glucosinolates which are established components of defence against herbivory. Nevertheless, additional factors may contribute to the inducibility of resistance, such as priming (van Hulten *et al.*, 2006; Ahmad *et al.*, 2011), epigenetic modifications (Rasmann *et al.*, 2012), or post-transcriptional effects (Gfeller *et al.*, 2011; Savchenko *et al.*, 2013). A study with a larger number of accessions and defence traits might be needed to explain the mechanistic aspects of the trade-off between constitutive and induced defences.

## Supplementary data

Supplementary data can be found at *JXB* online.

Supplementary Table S1. Genes known to be inducible after chewing herbivore attack.

Supplementary Table S2. Constitutive expression of genes involved in jasmonate, and glucosinolate biosynthesis and regulation.

Supplementary Table S3. Resistance experiment data of seven *A. thaliana* accessions against insect herbivores.

Supplementary Table S4. Glucosinolate levels in four *Arabidopsis* accessions.

Supplementary Table S5. List of primers for qPCR analyses.

Supplementary Fig. S1. Results of 10 000 Monte Carlo correlations between eight random gene and constitutive resistance across seven *Arabidopsis* accessions.

Supplementary Data
