# Supplementary material for: Trade-off between constitutive and inducible resistance against herbivores is only partially explained by gene expression and glucosinolate production
Source: J Exp Bot. 2015 Feb 25;66(9):2527–34. doi: 10.1093/jxb/erv033 (PMC4986863; doi:10.1093/jxb/erv033)
Supplement: Supplementary Data [file supp_66_9_2527__index.html]

Trade-off between constitutive and inducible resistance against herbivores is only partially explained by gene expression and glucosinolate production — Trade-off between constitutive and inducible resistance against herbivores is only partially explained by gene expression and glucosinolate production — Supplementary Data 

# Trade-off between constitutive and inducible resistance against herbivores is only partially explained by gene expression and glucosinolate production

## Supplementary Data

Data files

**Files in this Data Supplement:**

- Supplementary Data - Supplementary Data
