# Supplementary material for: Trade-off between constitutive and inducible resistance against herbivores is only partially explained by gene expression and glucosinolate production
Source: J Exp Bot. 2015 Feb 25;66(9):2527–34. doi: 10.1093/jxb/erv033 (PMC4986863; doi:10.1093/jxb/erv033)
Supplement: Supplementary Data [file supp_erv033_jexbot138784_file001.pdf]

Trade-off between constitutive and inducible resistance against herbivores is only partially explained by gene expression and glucosinolate production

Sergio Rasmann, Estelle Chassin, Julia Bilat, Gaétan Glauser, Philippe Reymond

Supplementary information

Table S1. Genes known to be inducible after chewing herbivore attack in Arabidopsis. Those genes were used for the classification of 34 Arabidopsis accessions based on constitutive gene expression levels, and the selection of the seven accessions used for the experiments.

Function	AGI	Short Description (TAIR10)
Glucosinolates	At4g39950	CYP79B2 (cytochrome P450)
	At1g74100	SOT16 (sulfotransferase)
	At5g60890	MYB34 (transcription factor)
	At3g16390	NSP3 (nitrile specifier protein 3)
	At1g52030	MBP2 (myrosinase-binding protein)
	At2g39330	Jacalin lectin family protein, myrosinase-associated protein
	At3g16470	Jacalin lectin family protein, myrosinase-associated protein
	At1g54000	GDSL-like lipase, myrosinase-associated protein
JA synthesis and signalling	At1g17420	LOX3 (lipoxygenase)
	At1g72520	LOX4 (lipoxygenase)
	At5g07010	ST2A (hydroxyjasmonate sulfotransferase)
	At5g13220	JAZ10 (jasmonate-ZIM-domain protein)
JA marker	At5g44420	PDF1.2 (low-molecular-weight cysteine-rich 77)
	At1g12240	Vacuolar invertase betaFruct4
	At2g24850	TAT3 (tyrosine aminotransferase 3); transaminase
	At5g24420	Glucosamine/galactosamine-6-phosphate isomerase-related

Table S2. Constitutive expression of genes involved in glucosinolate biosynthesis and regulation, and in jasmonate biosynthesis, signaling, and response, for seven Arabidopsis accessions.

Type	AGI ID	C-24	Kin-0	SH	HR-5	Col-0	ND-1	Ms-0
Glucosinolates	At4g39950	254.77	635.1	524.33	371.7	285.2	242.6	201.5
	At1g74100	704.87	706.67	1017.97	632.4	450.37	681.07	492
	At5g60890	212.67	169.9	203.87	264.6	226.37	143.7	120.7
	At3g16390	2087.93	1667.5	1149.27	747.6	1601.1	1325.47	970.5
	At1g52030	12.33	28.83	309.37	24.3	28.63	40.3	49.6
	At2g39330	15.23	20.57	15.37	7.2	16.43	10.83	20.9
	At3g16470	808.47	582.3	819.9	1048.4	632.03	725.13	358.3
	At1g54000	2079.9	1536.83	1370.87	1780.7	1459.83	1096.13	860.1
JA synthesis	At1g17420	55.43	19.1	36.73	23.9	42.43	45.87	29.5
	At1g72520	10.73	8.97	29.23	13.3	14.03	13.43	6.2
	At5g07010	12.3	12.5	12.83	11.1	6.07	9.93	22
	At5g13220	69.63	86.2	58.57	91.1	54.2	64	50.6
JA marker	At5g44420	9.07	3.17	15.17	28.5	15	8.23	2
	At1g12240	659.97	944.67	659.53	931.9	811.13	684.23	444.3
	At2g24850	28.4	20.43	18.57	27.8	24.6	20.43	25.6
	At5g24420	94.47	104.1	222.87	153.4	141.03	323.43	196.1
Average		541	483	447	406	435	397	283

Table S3. Constitutive (control treatment) and induced (*S. littoralis* and MeJA treatment) resistance of seven *A. thaliana* accessions against the specialist caterpillar *P. brassicae*, and the generalist caterpillar *S. littoralis*. Data represent averages (± SE) caterpillar dry weight.

Induction treatment	Accession	*P. brassicae* (mg)	*S. littoralis* (mg)
Control	C-24	0.899 +/- 0.024	0.199 +/- 0.026
	Col-0	0.594 +/- 0.029	0.26 +/- 0.013
	HR-5	0.826 +/- 0.086	0.254 +/- 0.01
	Kin-0	0.725 +/- 0.03	0.235 +/- 0.01
	Moscow-0	0.922 +/- 0.026	0.42 +/- 0.017
	ND-1	0.784 +/- 0.024	0.582 +/- 0.057
	SH	0.573 +/- 0.065	0.211 +/- 0.006
S. littoralis	C-24	0.794 +/- 0.045	0.201 +/- 0.023
	Col-0	0.427 +/- 0.034	0.234 +/- 0.011
	HR-5	0.482 +/- 0.053	0.233 +/- 0.005
	Kin-0	0.714 +/- 0.05	0.157 +/- 0.014
	Moscow-0	0.681 +/- 0.034	0.322 +/- 0.028
	ND-1	0.636 +/- 0.022	0.359 +/- 0.014
	SH	0.808 +/- 0.037	0.175 +/- 0.015
MeJA	C-24	0.971 +/- 0.016	0.272 +/- 0.03
	Col-0	0.619 +/- 0.066	0.257 +/- 0.013
	HR-5	0.628 +/- 0.021	0.284 +/- 0.041
	Kin-0	0.376 +/- 0.061	0.178 +/- 0.003
	Moscow-0	0.869 +/- 0.043	0.265 +/- 0.023
	ND-1	0.744 +/- 0.034	0.335 +/- 0.03
	SH	0.605 +/- 0.01	0.209 +/- 0.006

Table S4. Glucosinolate levels in four Arabidopsis accessions

	Col-0		HR-5		Ms-0		Nd-1	
**Glucosinolate**	**Control**	**Induced**	**Control**	**Induced**	**Control**	**Induced**	**Control**	**Induced**
2-propenyl	0 +/- 0	0 +/- 0	0.017 +/- 0.006	0.024 +/- 0.004	0.839 +/- 0.244	0.743 +/- 0.140	0.005 +/- 0.001	0.005 +/- 0.003
3-hydroxypropyl	0.001 +/- 0.001	0 +/- 0	0.275 +/- 0.172	0.088 +/- 0.083	0 +/- 0	0.001 +/- 0.001	0.871 +/- 0.113	1.191 +/- 0.209
7-methylthioheptyl (7MTH)	0.026 +/- 0.002	0.027 +/- 0.001	0.050 +/- 0.011	0.059 +/- 0.012	0.025 +/- 0.003	0.033 +/- 0.010	0.020 +/- 0.002	0.023 +/- 0.002
8-methylthiooctyl (8MTO)	0.064 +/- 0.005	0.051 +/- 0.004	0.177 +/- 0.017	0.188 +/- 0.037	0.216 +/- 0.042	0.194 +/- 0.019	0.130 +/- 0.016	0.150 +/- 0.018
glucobrassicanapin	0 +/- 0	0 +/- 0	0.033 +/- 0.011	0.041 +/- 0.008	0 +/- 0	0.006 +/- 0.006	0 +/- 0	0 +/- 0
glucobrassicin (I3M)	0.181 +/- 0.027	0.586 +/- 0.163	0.154 +/- 0.017	0.203 +/- 0.015	0.117 +/- 0.025	0.226 +/- 0.030	0.228 +/- 0.027	0.424 +/- 0.080
glucoerucin (4MTB)	0.190 +/- 0.026	0.132 +/- 0.019	0.003 +/- 0.002	0.004 +/- 0.002	0 +/- 0	0 +/- 0	0 +/- 0	0 +/- 0
glucohirsutin (8MSOO)	0.116 +/- 0.017	0.106 +/- 0.010	0.334 +/- 0.040	0.344 +/- 0.055	0.534 +/- 0.128	0.397 +/- 0.041	0.272 +/- 0.058	0.311 +/- 0.070
glucoiberin (3MSOP)	0.103 +/- 0.014	0.161 +/- 0.022	0.023 +/- 0.014	0.028 +/- 0.028	0.034 +/- 0.008	0.032 +/- 0.006	0.122 +/- 0.017	0.187 +/- 0.032
gluconapin	0.001 +/- 0.001	0 +/- 0	0.344 +/- 0.117	0.449 +/- 0.099	0.029 +/- 0.006	0.094 +/- 0.068	0 +/- 0	0 +/- 0
glucoraphanin (4MSOB)	0.715 +/- 0.1	1.200 +/- 0.172	0.008 +/- 0.004	0.008 +/- 0.002	0 +/- 0	0.001 +/- 0.001	0.020 +/- 0.002	0.025 +/- 0.004
glucotropeolin	0 +/- 0	0 +/- 0	0 +/- 0	0 +/- 0	0 +/- 0	0 +/- 0	0 +/- 0	0 +/- 0
methoxyglucobrassicin (4MOI3M)	0.019 +/- 0.001	0.025 +/- 0.003	0.015 +/- 0.001	0.016 +/- 0.001	0.020 +/- 0.002	0.020 +/- 0.001	0.012 +/- 0	0.015 +/- 0.001
neoglucobrassicin (1MOI3M)	0.003 +/- 0.002	0.039 +/- 0.010	0 +/- 0	0 +/- 0	0.004 +/- 0.003	0.011 +/- 0.002	0 +/- 0	0 +/- 0
progoitrin isomer	0 +/- 0	0 +/- 0	0.097 +/- 0.032	0.130 +/- 0.028	0 +/- 0	0.045 +/- 0.045	0 +/- 0	0 +/- 0
progoitrin	0.001 +/- 0.001	0 +/- 0	0.302 +/- 0.101	0.405 +/- 0.088	0 +/- 0	0.143 +/- 0.143	0 +/- 0	0 +/- 0
TOTAL	1.420 +/- 0.197	2.327 +/- 0.404	1.832 +/- 0.545	1.987 +/- 0.462	1.818 +/- 0.461	1.946 +/- 0.513	1.680 +/- 0.236	2.331 +/- 0.419

Values (μmol/g FW) are the mean (±SE) of 6 measurements

Table S5. List of primers used in this study

AOC2 (At3g25770)	Fwd	5'-CACGTCCCAGAGAAGAAAGG-3'
	Rev	3'-CGAGGAACGAATCCTCGTAA-3'
CYP79B3 (At2g22330)	Fwd	5'-CTTTGCTTACCGCTGATGAA-3'
	Rev	5'-GCGTTTGA TGGGTTGTCTG-3'
VSP2 (At5g24770)	Fwd	5'-GGTGCCCGCAAATTGCAAAGACTA-3'
	Rev	5'-GGTTGATGCTCCGGTCCCTAACCA-3'
SAND (At2g28390)	Fwd	5'-AACTCTATGCAGCATTTGATCCACT-3'
	Rev	5'-TGATTGCATATCTTTATCGCCATC-3'

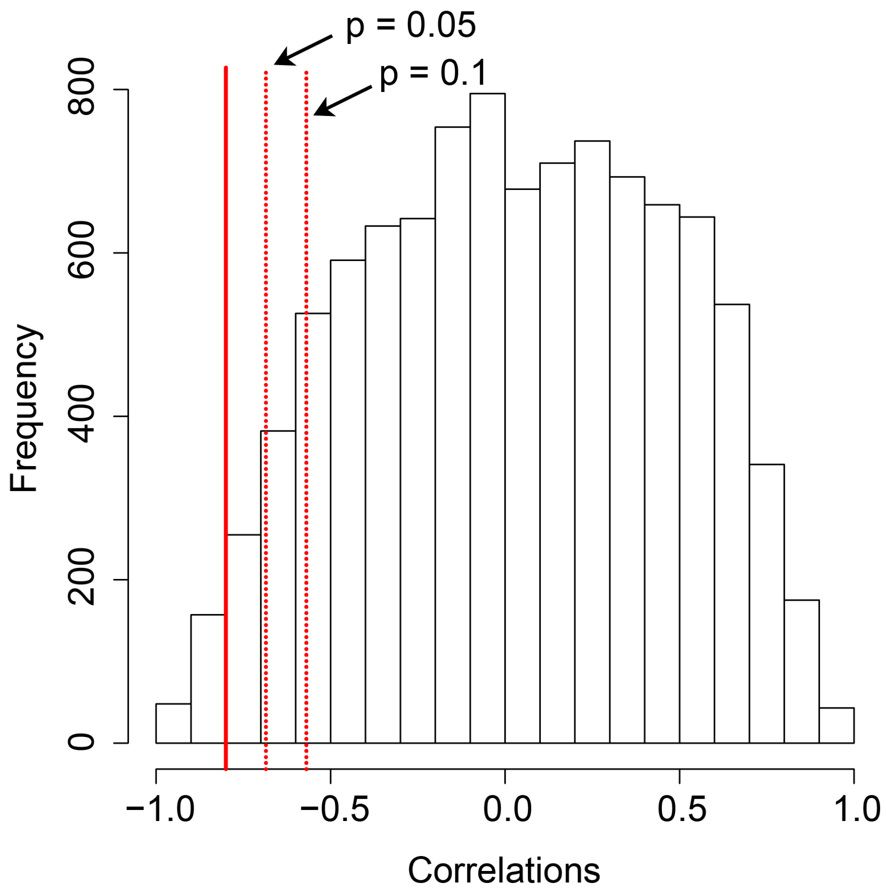

Figure S1. Selection of random genes. Shown is the histogram of correlations between the average of 10 randomly selected genes and the constitutive resistance against *S. littoralis* across seven Arabidopsis accessions. Solid line indicates the correlation coefficient for the 8 genes related to glucosinolate production. Dotted lines represent the 10% and 5% quantile for the 10000 correlations using random gene selection.
